# DEK-NUP214融合基因阳性高危急性髓系白血病眼睑浸润1例

**DOI:** 10.3760/cma.j.cn121090-20231207-00297-1

**Published:** 2024-05

**Authors:** 小珍 李, 睿 黄, 天琦 高, 秀雯 谢, 艳群 周, 清莉 曾, 基铎 刘, 学奎 古, 增慧 刘

**Affiliations:** 1 广州中医药大学第一附属医院，广州 510405 the First Affiliated Hospital of Guangzhou University of Chinese Medicine, Guangzhou 510405, China; 2 广州中医药大学，广州 510405 Guangzhou University of Chinese Medicine, Guangzhou 510405, China; 3 南方医科大学珠江医院血液科，广州 510282 Department of Hematology, Zhujiang Hospital of Southern Medical University, Guangzhou 510282, China; 4 贵州中医药大学，贵阳 550025 Guizhou University of Chinese Medicine, Guiyang 550025,China

患者，男，15岁，2021年12月23日因“反复发热1个月，右眼睑进行性红肿4 d”入院。入院时高热，热峰至39.8 °C，重度贫血貌，胸骨压痛，左眼睑红肿，无压痛，视力正常。血常规：WBC 26.82×10^9^/L，中性粒细胞绝对计数15.15×10^9^/L，HGB 121 g/L，PLT 393×10^9^/L。骨髓象提示急性粒细胞白血病未分化型（AML-M_1_）。免疫分型：原始幼稚细胞群A约87.4％，表达CD43、CD38；部分表达MPD、HLA-DR、CD11b、CD34、CD117、CD123。骨髓活检病理符合急性髓系白血病病理改变。多重PCR筛查56种白血病相关融合基因：t（6;9）（p23;q34）/DEK-NUP214融合基因阳性（突变比例272.27％）；髓系相关突变二代测序（NGS）：FLT3-ITD突变阳性（AR＝0.759），RUNX1突变阳性（p.Asp332Asn突变比例46.9％）、WT1突变阳性（p.Arg385fs突变比例15.0％和p.Arg375fs突变比例14.9％）。眼眶、视神经MR平扫+增强：鼻周、左侧眶周及左侧颞部皮下异常信号，符合白血病髓外病灶影像。在使用羟基脲降细胞处理后予标准剂量DA方案诱导治疗。同时经验性予亚胺培南和利奈唑胺抗感染治疗。诱导治疗后，患者三系快速下降，然而体温仍升高，眼睑部位较前进展。上眼睑出现水疱，眶周和鼻周肿胀，病灶逐渐累及左下眼睑、右上眼睑、右下眼睑和双侧眶周区域。2022年1月4日眼眶、视神经MRI平扫+增强：鼻周、双侧眶周及颞部皮下异常信号，符合白血病髓外病灶，左侧较前略减轻，右侧较前略明显。基于患者眼睑病灶进展，考虑此前方案疗效不佳。2021年12月31日在DA方案基础上加用维奈托克继续诱导治疗。患者处于粒细胞缺乏状态，服用泊沙康唑预防真菌感染，调整维奈托克剂量为50 mg/d，持续2周。患者在DAV方案诱导治疗结束后眼睑病灶基本消退，化疗后出现Ⅳ级骨髓抑制，粒细胞缺乏期持续21 d。骨髓原始粒细胞占3.5％，见吞噬细胞及吞噬血细胞现象。2022年1月31日始予第2个疗程诱导治疗，调整为IAV方案：伊达比星12 mg/m^2^第1～3天，阿糖胞苷100 mg/m^2^第1～7天（间隔12 h静脉滴注），维奈托克50 mg/d第1～14天，并鞘内注射甲氨蝶呤15 mg、阿糖胞苷50 mg。脑脊液检查未见中枢神经系统浸润。2个疗程诱导化疗后复查骨髓：增生活跃，未见原始幼稚细胞；流式细胞术检测微小残留病（MRD）：分析见原始幼稚细胞群约0.23％，表达CD13、CD15、CD16、CD34、CD117、CD123、HLA-DR，部分表达CD33、CD64。复查DEK-NUP214融合基因突变比例0.46％，FLT3-ITD突变阴性，WT1突变比例0.08％。此后患者接受中大剂量阿糖胞苷巩固治疗，行3次腰椎穿刺鞘内注射。4月7日复查骨髓象：增生低下，未见原始幼稚细胞；流式细胞术检测MRD阴性。复查DEK-NUP214融合基因突变比例0.08％，FLT3-ITD突变阴性，WT1突变比例0.12％。眼眶、视神经MRI平扫+增强：鼻周、双侧眶周及颞部皮下异常信号基本消失。患者在3个疗程化疗后接受单倍体造血干细胞移植。2022年4月20日进行含氟达拉滨、白消安和环磷酰胺的FBUCY预处理，分别于4月27日和4月28日输注其父的造血干细胞，单个核细胞7.23×10^8^/kg，CD34^+^细胞5.89×10^6^/kg。持续服用环孢素A和霉酚酸酯用于预防移植物抗宿主病。移植后第17天粒细胞植入，第19天血小板植入。移植后1个月，复查骨髓、MRD、基因结果均为阴性。患者处于完全缓解状态1年余，后因重症肺炎死亡。

